# Acoustic deterrence as a mitigation tool for seal-human conflicts in the Helgoland Dune swimming zone

**DOI:** 10.1038/s41598-025-17236-2

**Published:** 2025-09-15

**Authors:** Kim Nina Heimberg, Stephanie Gross, Abbo van Neer, Juan Felipe Escobar-Calderon, Andreas Ruser, Tobias Schaffeld, Ursula Siebert

**Affiliations:** Institute for Terrestrial and Aquatic Wildlife Research, University of Veterinary Medicine Hannover, Foundation, Werftstraße 6, 25761 Buesum, Germany

**Keywords:** Conservation biology, Marine mammals, Marine biology, Animal behaviour

## Abstract

Successful species conservation and rising tourism in coastal ecosystems lead to more human-wildlife conflicts. Non-invasive conflict mitigation measures are needed to ensure human safety and wildlife conservation. The Helgoland Dune, a small German offshore island, is home to Germany’s largest grey seal (*Halichoerus grypus*) colony alongside popular tourist beaches, leading to frequent human-seal encounters. Previous physical incidents in the shared swimming zone have raised safety concerns. This study evaluated an acoustic deterrent system (ADS) using Targeted Acoustic Startle Technology (TAST) to reduce such interactions. During two field trials, the ADS emitted pulsed signals (2 s, 700–1500 Hz, 180 dB re 1 µPa) while a camera tracked seal movements. Analysing 22,086 positions via a cumulative link mixed model (CLMM) revealed seals were 3.57 times more likely (odds ratio, CI: 2.38–5.37, p-value < 0.001) to occupy the two farther distance categories (10–25 m, > 25 m) during signal emission, compared to the closest distance zone (< 10 m). Individual variability accounted for 94% (ICC) of behavioural differences. Results provide the first empirical evidence of the ADS’s effectiveness in a recreational context as a non-invasive deterrent tool, while maintaining seal access to surrounding habitat crucial to them.

**Supplementary Information:**

The online version contains supplementary material available at 10.1038/s41598-025-17236-2.

## Introduction

As tourism, recreation, and other anthropogenic activities increasingly encroach upon marine environments, interactions between humans and wildlife in coastal regions are becoming more frequent and complex^1–3^. This trend is further intensified by the recovery of wildlife populations, particularly in areas where human activities overlap with critical habitats^4,5^. While these interactions provide valuable opportunities for wildlife observation and education, they also raise concerns regarding human safety, animal welfare, and the disturbance of natural behaviours^6–10^.

A striking example of this dynamic is the grey seal (*Halichoerus grypus*) colony in the Wadden Sea as part of the wider North Sea population, which faced virtual extirpation by the late Middle Ages due to intensive hunting and human encroachment^11^. Following a gradual implementation of hunting bans in the last century, grey seals began to recolonise the southern North Sea^11–13^. Small colonies established in the German and Dutch Wadden Sea and expanded significantly due to protection efforts and immigration from the UK^11,14^. Despite this recovery, grey seals remain classified as endangered on the German Red List, as their numbers are still far below historical levels^15^.

The Helgoland Dune, located offshore in the North Sea, has become a critical habitat for the grey seal population of the North Sea, hosting the largest colony in German waters. Annual coordinated surveys conducted since 2008 indicate steady growth in this colony, with consistently rising birth rates each year^16^. Regular sightings have been recorded since 1989^17^, and the first birth was documented in 1996^18^. Today, the Helgoland Dune is year-round inhabited by all age classes, serving as a breeding and resting site, with grey seal numbers peaking during pupping as well as moulting season^12,19^. However, the island’s popularity as a tourist destination has led to significant overlap between human and grey seal habitats, resulting in frequent close encounters on land and in the water^20^.

Locally, land-based interactions are managed through measures such as minimum approach distances, wildlife resting zones, and visitor guidelines^21^. However, water-based interactions remain largely unregulated, posing a significant challenge for coexistence^20,21^. To enhance safety and reduce disturbance by humans, the beach has been spatially divided into designated bathing and non-bathing zones. Despite this separation, seals frequently enter swimming areas, leading to close encounters with swimmers^20^. This issue is particularly acute in the south beach’s swimming zone, where limited space and increasing interactions have raised safety concerns, especially following incidents of direct contact initiated by seals, such as touching, grabbing, or scratching^20^. Whereas current management strategies have already shown some positive effects, there is still the need for novel, non-invasive solutions to ensure both human safety and seal conservation^21^. Importantly, for grey seals, this area serves primarily as a resting and socialising spot, not a foraging ground. This suggests a lower foraging-driven motivation for being in the area compared to contexts like fisheries depredation. Nonetheless, other factors, such as curiosity, habituation to human presence, or social dynamics, may still motivate individuals to enter or remain in these human-used spaces^22–24^.

Building on these challenges, the presented study investigates the potential of an acoustic deterrent device to modify grey seal behaviour in an area where human activities intersect with their natural habitat^20,25^. While traditional acoustic deterrents have been widely used in marine environments, particularly in fisheries and conservation contexts, their application in managing human-seal interactions in recreational waters remains largely untested^26,27^. Specifically, the study evaluates the effectiveness of a novel acoustic deterrent system (ADS) utilising Targeted Acoustic Startle Technology (TAST), an advanced acoustic tool designed to elicit an involuntary startle response in the target species^28^. Importantly, in grey seals, the startle reflex typically manifests as a flight response, which is the critical behaviour targeted for deterrence in this context^29^. Unlike traditional acoustic deterrents that rely on continuous and aversive noise, the ADS emits brief, low-frequency pulsed signals at low duty cycles, specifically tailored to the auditory sensitivity of grey seals^27,30^. This targeted approach is intended to trigger the seal’s innate acoustic startle reflex, thereby deterring them while reducing the risk of habituation and minimising impacts on non-target species^25,29^. By focusing on species-specific auditory mechanisms, the ADS represents a significant advancement in acoustic deterrent technology, offering a more precise and ecologically considerate solution^25,29,30^.

While the ADS has previously demonstrated effectiveness in reducing seal depredation at fish farms and in other prey-motivated contexts^25,30–33^the novelty and promise of this study lie in testing the startle-based ADS in a setting where strong prey motivation is absent. Here, grey seals are less motivated by foraging, which may enhance the deterrent’s success by primarily targeting non-foraging behaviours such as curiosity or habituation to human presence.

This study aimed to assess the suitability of the ADS as a non-invasive tool for mitigating human-seal conflicts in shared coastal waters, specifically by deterring grey seals from high-use swimming areas while minimising ecological disruption. Although swimmers were present in the designated swimming area during the observation periods, their numbers were lower than during peak tourist season in mid to late summer. The study focused exclusively on seal behaviour in response to the acoustic signal, human reactions or perceptions were not assessed. Importantly, the ADS was not aimedat deterring or influencing people, but rather to reduce the frequency and proximity of grey seal approaches to swimmers. This moderate level of human activity provided a realistic shared-use environment, allowing for focused and controlled observations of seal behaviour during this initial field test of the ADS in a recreational setting.

Two field trials were conducted by employing the ADS in the swimming zone at the Helgoland Dune’s southern beach. An underwater transducer broadcasted ADS signals, while a high-resolution camera system simultaneously recorded grey seal movements. Seal positions extracted from the camera footage were analysed to determine whether seals were observed farther from the transducer during signal emissions compared to emission-free control periods, suggesting a potential avoidance or deterrence reaction. By evaluating whether the ADS signals effectively deter seals from the restricted area without causing full displacement, this study provides the first empirical assessment of the ADS as a non-invasive tool for reducing human-seal encounters in shared coastal environments, offering a novel approach to promote coexistence.

## Materials and methods

### Study area and data collection periods

**Fig. 1:**
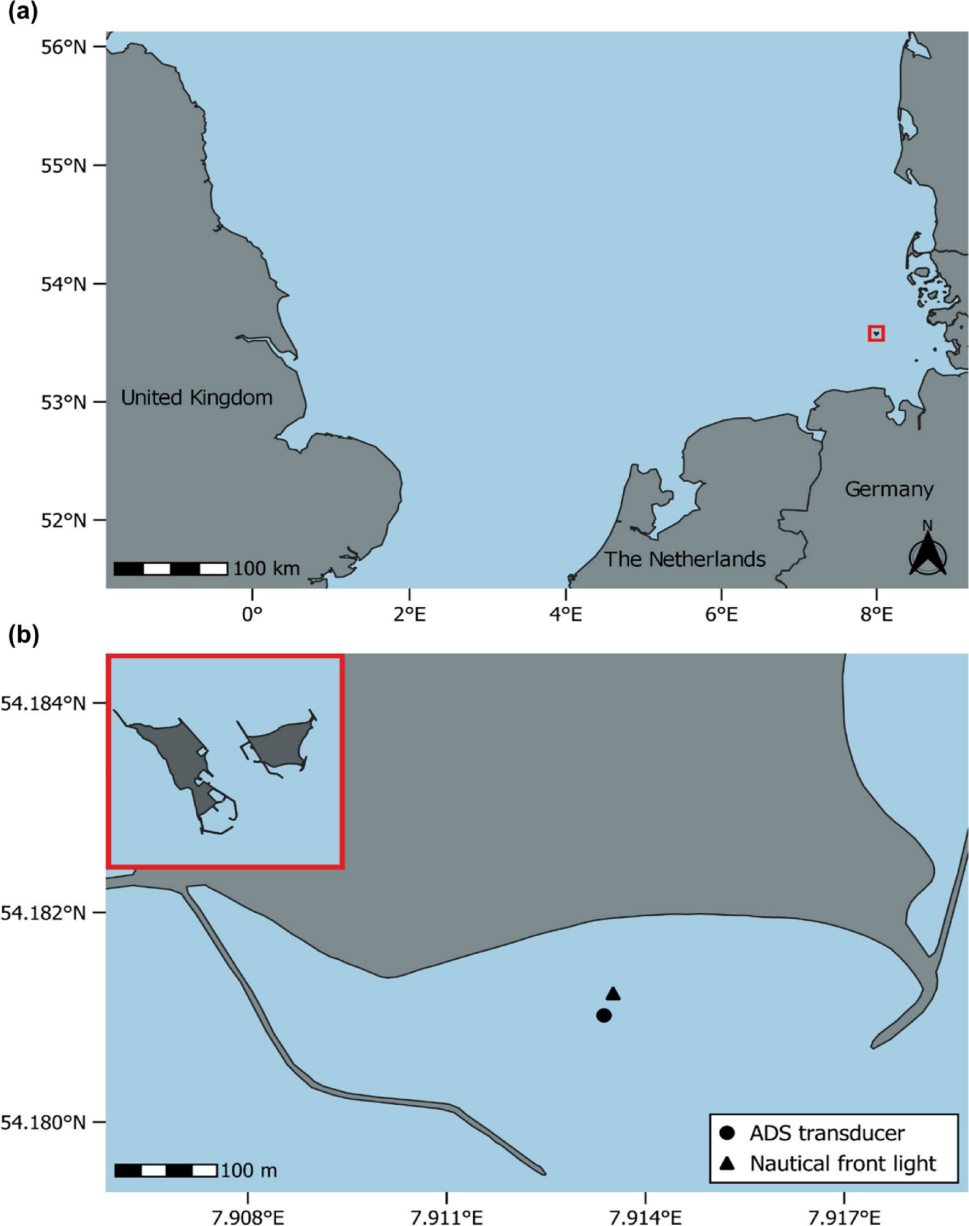
Location of the study area in the southeastern North Sea. (**a**) Overview map of Northwestern Europe highlighting the position of the Helgoland archipelago (red box) off the German North Sea coast. (**b**) The red box provides an overview of the main island of Helgoland (left) and the adjacent Helgoland Dune (right). The larger map section shows the southern beach swimming zone on the Helgoland Dune, where the field experiment was conducted. The position of the acoustic deterrent system (ADS) transducer unit is marked with a black circle, and the nearby nautical front light is indicated by a black triangle. Maps were created using QGIS 3.34, available at https://qgis.org.

The study was conducted over two independent field trial periods: the first from June 22 to 28, 2023 (trial period 1), and the second from May 13 to 17, 2024 (trial period 2). Both field trials took place on the Helgoland Dune (54° 11′ 5” N, 7° 54′ 44” E), a small island (0.7 km²) in the North Sea, approximately 55 km from the German mainland (Fig. 1a). The Helgoland Dune is part of the Helgoland archipelago, located 750 m east of the main island of Helgoland. Research focused on the southern beach swimming zone (Fig. 1b), characterised by shallow water conditions, ranging approximately from 1 to 3 m in depth, with tidal variations causing strong fluctuations. During low tide, water can be occasionally absent near the shore. This area is a high-traffic zone for grey seals, leading to frequent human-seal interactions. Human swimmers were present in the designated zone during trial periods, albeit in reduced numbers relative to peak tourist season (mid to late summer). This moderate level of human activity allowed for focused observation of seal behaviour under realistic, shared-use conditions.

### Experimental setup

#### Acoustic deterrent system

The acoustic deterrent system (ADS) consisted of a control unit (GenusWave Ltd, Scotland, United Kingdom), a unit of two acoustic underwater transducers (Lubell LL9161, Lubell Labs Inc., USA), and an external power source (12 V 60Ah Gel Deep Cycle Battery BAT412550104, Victron Energy B.V., The Netherlands). The transducer unit was mounted on a custom-built metal rack and suspended at a water depth of approximately 1 m, positioned about 25 m southwest of the nautical front light within the southern beach swimming zone (Fig. 1b). A hydrophone (SoundTrap 300HF, Ocean Instruments, New Zealand) was attached to the metal rack to record the temporal sound activity of the ADS. The control unit and the external power source were positioned outside the water, on the concrete base of the nautical front light to remain accessible. The tested acoustic stimulus consisted of band-limited noise (0.2 s duration, 700–1500 Hz frequency range centred at 1000 Hz) emitted at a source level of approximately 180 dB re 1 µPa, with a rise time of < 5 ms. Sound pulses were pre-programmed to emit at irregular, pseudo-random intervals (2–28 s), maintaining a duty cycle of 2%. The system was activated daily at 9:00 AM and deactivated at 6:00 PM using a synchronised clock to ensure precise timing and consistency throughout the trial periods. During operation, the system automatically alternated between ON and OFF phases. During ON phases, the ADS emitted acoustic signals, whereas OFF phases served as control periods without sound emissions. In the first trial period, ON and OFF phases lasted 42 and 58 min, respectively. In the second trial period, the duration of both ON and OFF phases was adjusted to 45 min to optimise the operational schedule and balance exposure and control periods.

#### Visual observation of grey seals

To record potential behavioural responses of the grey seals, a camera (GoPro HERO 11, GoPro Inc., CA, USA) was mounted in an elevated position (approx. 11 m high, on the upper platform of the nautical front light), providing an unobstructed view of the area around the transducer unit. The camera was powered by an external power bank (Litionite Tanker 90 W, 50,000 mAh, Litionite S.R.L., Italy) connected to a keep-alive load (QCCP-Adapter, Timelapse Production Black Forest, Germany) preventing the camera from complete shutdown. The power bank was further connected to an external power source to ensure continuous operation. The camera was configured using GoPro Labs firmware (GoPro Labs custom firmware, V 2.10.70, GoPro Inc., CA, USA) to capture 5 K timelapse videos (4:3 aspect ratio). A wide field of view was selected to comprehensively cover the area around the transducer unit. The camera took single photos at a 2-second resolution. Daily recordings were synchronised with the visible and manual activation and deactivation of the ADS system, ensuring alignment with the camera’s time schedule at the beginning and end of each day.

### Data processing

All data processing and statistical analysis were conducted in R 4.4.0^34^. Audio recordings from the hydrophone (sampling rate: 44.1 kHz, 16 bit) were processed using a custom-built workflow. Only recordings between 9:00 AM and 6:00 PM were retained to match the ADS’s operational period. Each session was divided into 10-minute intervals and signal peaks were detected using the package ‘pracma’^35^. Since the hydrophone was attached close to the transducer the received levels were close to or even exceeding the clipping level. This allowed for a detection by a peak finder, set to a minimum threshold of 25,000 digits, ensuring distinct signal events. A minimum separation of 1.9 s between peaks was enforced to prevent closely spaced events from being grouped incorrectly. To distinguish between signal emission and control periods, ON phases were identified by ensuring that the time interval between successive signals did not exceed 28 seconds. This approach ensured a clear distinction between ON and OFF phases. Due to battery recharging (from 10 AM on May 14, 2024, to 11 AM on May 15, 2024) and the transducer unit not being fully submerged during extreme low-tide events in the second trial period, the ADS was temporarily switched off. Such events were included as OFF phases, as no signal was emitted.

### Spatio-temporal categorisation of seal positions

**Fig. 2:**
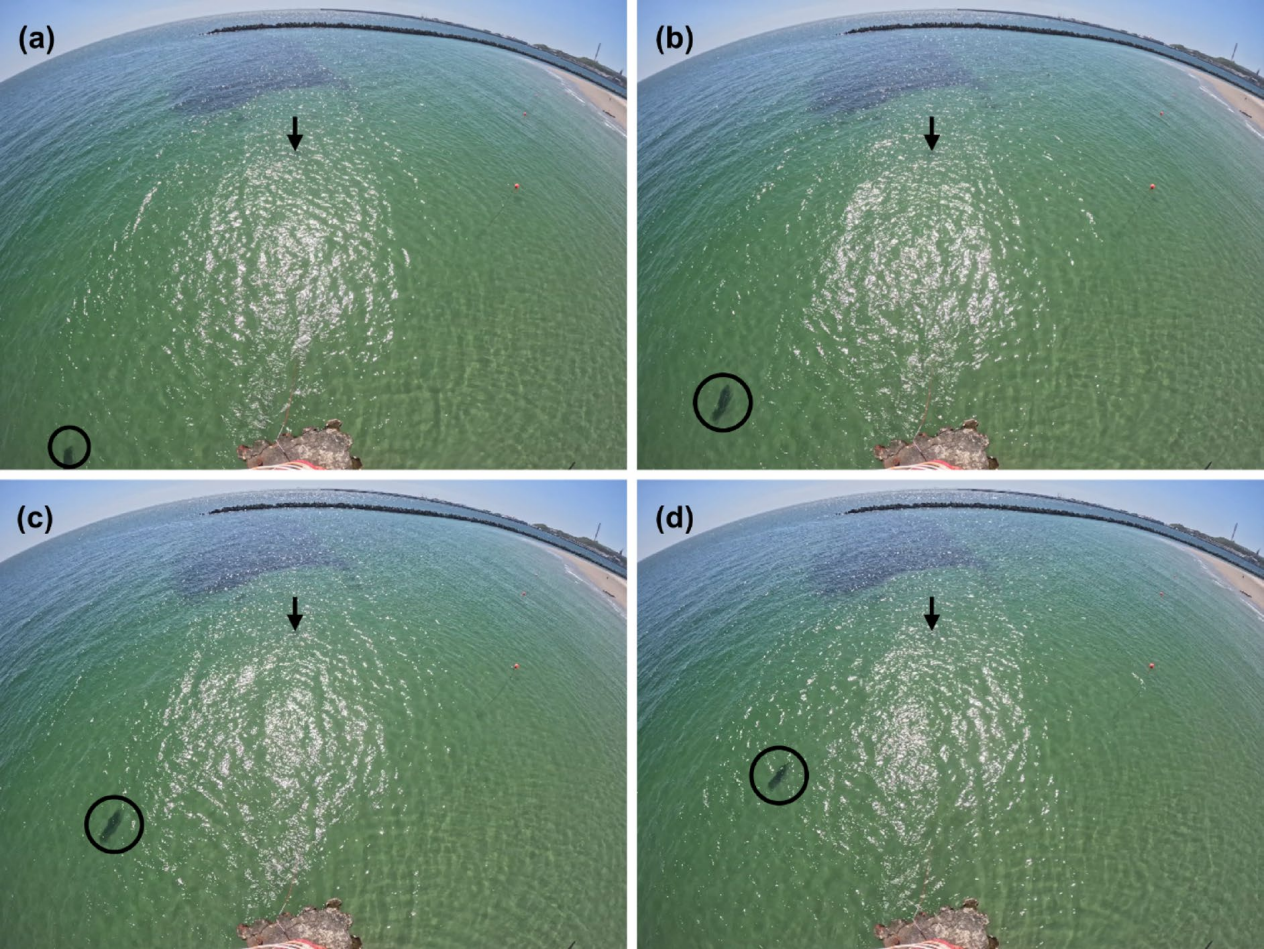
Representative of four raw still image frames (**a**-**d**) extracted from the timelapse video footage visualising the first four recorded positions of a grey seal track in the study area in the swimming zone of the Helgoland Dune’s southern beach. The images (**a**-**d**) are presented in strict chronological order, each separated by 2 s. In each frame, the tracked grey seal is visible diving with its position indicated by a black circle, demonstrating the visual quality of the data used for position extraction and illustrating the seal in situ prior to detailed analysis The position of the transducer unit is marked with a black arrow.

**Fig. 3:**
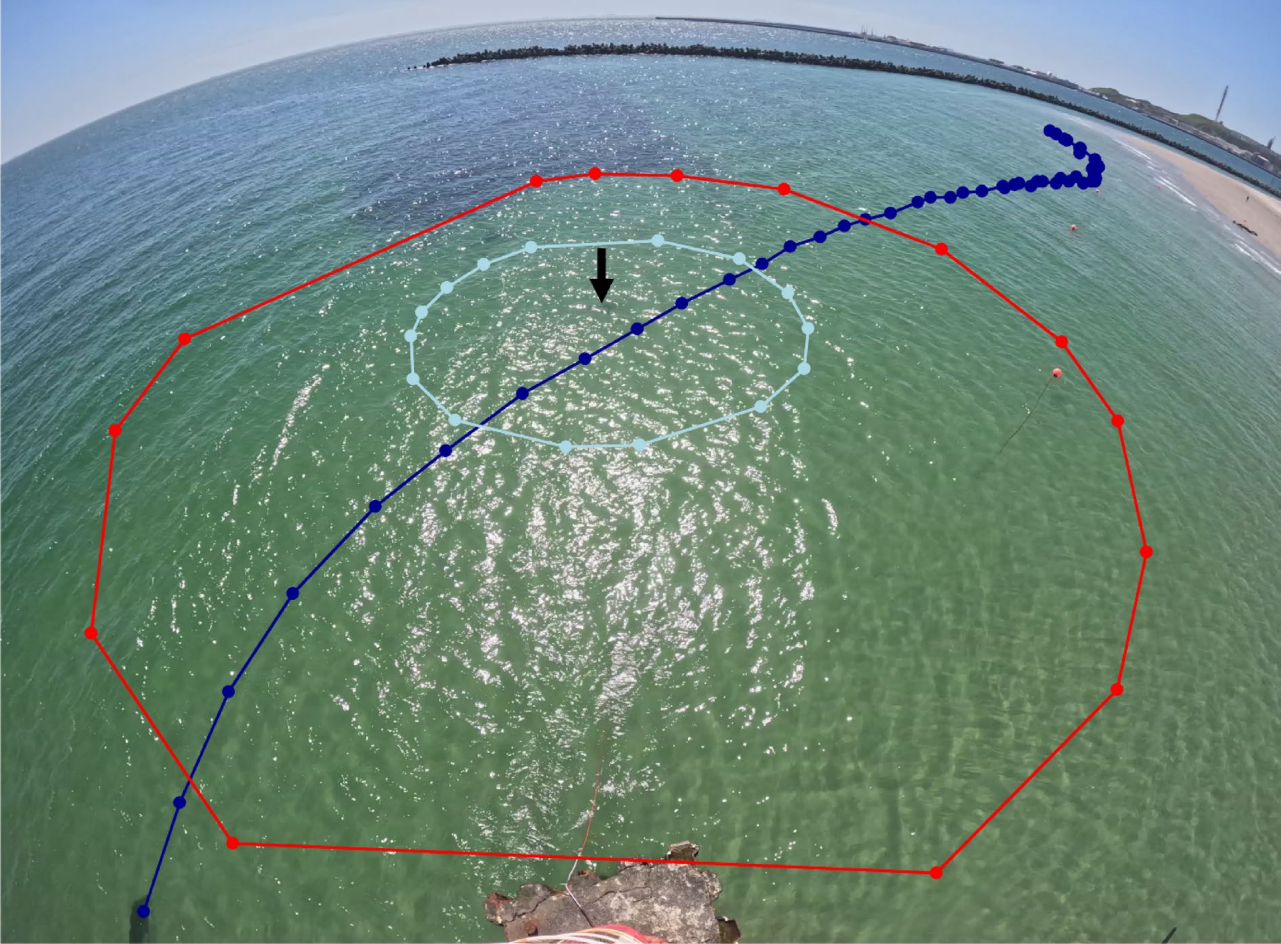
Illustration of grey seal tracking and defined distance zones. This extracted image shows the study area in the swimming zone of the Helgoland Dune’s southern beach. The position of the transducer unit is marked with a black arrow. The dark blue track represents a reconstructed movement track of a grey seal, with individual positional data points (dark blue dots) extracted from the recorded video footage. The tracked grey seal itself is visible at the first dark blue dot in the lower-left corner of the image. The light blue dots correspond to the positions of the 10 m distance markers, forming the light blue circle that indicates the outer border of the 10 m radius around the transducer. Similarly, the red dots correspond to the 25 m distance markers, outlining the red polygon that indicates the outer boarder of the 25 m radius around the transducer further marking the transition to the > 25 m distance zone.

Video footage was processed using the package ‘av’^36^. Individual still image frames were extracted using the function ‘av_video_images’. Each image was visually inspected by a trained observer to record the spatio-temporal occurrence of individual seals in the camera’s field of view. Representative still image frames illustrating this extraction process and the visual quality of the data are provided in Fig. 2. For each image with present seals, positions were manually extracted using the function ‘locator’^34^ within a custom-built workflow in R. The image pixel coordinates (x, y) of each position were recorded, along with a track ID and the corresponding timestamp. This process was repeated for all images to reconstruct unique movement tracks of seals, enabling detailed analysis of their behaviour and spatial movement patterns over time. An example of a reconstructed seal track in the defined study area is provided in Fig. 3.

During both trial periods, two distance radii (10 m and 25 m) around the transducer unit were measured, with the camera simultaneously recording corresponding distance markers. The distances were chosen based on expected deterrent ranges^29^ and practicability in-situ. Using the reported source level of 180 dB re 1 µPa, a response threshold of 159 dB re 1 µPa^29^ and a practical transmission loss factor of 15 dB, an effective distance of 25 m can be expected. The 10 m distance should represent a further intermediate-step, if effectiveness is lower than expected. Pixel coordinates representing the marker positions were extracted from the images using the function ‘locator’^34^ and converted into actual distance measurements. These coordinates were further transformed into spatial polygons using the ‘st_polygon’ and ‘st_sfc’ functions from the package ‘sf’^37,38^ creating spatial representations of three designated distance zones around the transducer unit (< 10 m, 10–25 m, > 25 m) (see also Fig. 3). The polygons were subsequently used to classify the recorded seal positions into the three distance categories via the ‘st_intersects’ function from the ‘sf’ package^37,38^. To assess the potential influence of the ADS signal on seal proximity to the transducer unit, each position was further cross-referenced with the corresponding signal phase (ON/OFF), allowing for an analysis of spatial distribution under the two different acoustic conditions.

### Data analysis

To assess the potential deterrent effect of the ADS signal on grey seal behaviour, a cumulative link mixed model (CLMM) was fitted using the function ‘clmm’ from the ‘ordinal’ package^39^. The CLMM was chosen to account for the ordinal nature of the response variable and to incorporate potential random effects, ensuring robust inference on the influence of the ADS signal. The model employed a ‘logit’ link function, assuming proportional odds, meaning the effect of the ADS signal is consistent across the three distance categories (< 10 m, 10–25 m, > 25 m). The Hessian matrix was computed to ensure accurate standard errors and p-values. A combined dataset comprising observations from both trial periods was analysed to increase sample size, improve robustness, and enhance the generalisability of the results. For this initial field test of the ADS in a recreational setting, the model structure was pre-defined and did not involve a process of fixed-effect variable selection. Our primary objective was to evaluate the direct deterrent effect of the ADS signal. Therefore, the ADS signal was included as the sole fixed-effect categorical predictor variable with two levels: OFF and ON. The response variable, distance, representing distance-classified seal positions, was defined as an ordered factor with three levels: < 10 m, 10–25 m, and > 25 m. To account for potential correlations within the data, track ID was included as a random intercept effect in the model. Each track ID uniquely identified a continuous movement sequence of recorded positions over time and was used to account for the temporal dependency of positions within a track. Although individual seals were not uniquely identified, this approach controlled for the non-independence of repeated measurements (successive positions) within the same track. It remains unknown whether the same individual may have been observed in multiple tracks, since seals could not be individually identified. Although factors such as time of day, ambient noise levels, individual prior exposure, or environmental variables (e.g., tidal state) might potentially influence seal responses, these were neither systematically varied nor recorded within the controlled experimental design of this study. In particular, temporal variables, such as time of day or progression within a trial session, were not included as fixed effects because the short and controlled duration of each session was designed to compare behavioural responses between signal ON and OFF phases in homogenous conditions, rather than to assess behavioural trends over time. To quantify the proportion of variance in seal distances explained by individual differences, the adjusted intraclass correlation coefficient (ICC) was calculated using the function ‘icc’ from the package ‘performance’^40^. Additionally, the odds ratio for the fixed effect of the ADS signal phase (ON vs. OFF) and its 95% confidence interval (CI) were obtained using the function ‘tab_model’ from the package ‘sjPlot’^41^. This odds ratio represents the relative likelihood of a seal being recorded in a higher distance category (10–25 m or > 25 m) when the ADS signal is ON compared to when it is OFF. To further interpret model results, threshold coefficients estimated by the model were converted into cumulative probabilities using the inverse logit function:

1 $$\:\text{P}\left(\text{Y}\le\:\text{d}\text{i}\text{s}\text{t}\text{a}\text{n}\text{c}\text{e}\right)=\frac{1}{1+{\text{e}}^{-\:\text{t}\text{h}\text{r}\text{e}\text{s}\text{h}\text{o}\text{l}\text{d}\:\text{c}\text{o}\text{e}\text{f}\text{f}\text{i}\text{c}\text{i}\text{e}\text{n}\text{t}}}$$

In this Eq. (1), P(Y ≤ distance) represents the probability that the seal’s position falls into a specific distance category (or a closer one). The threshold corresponds to the boundary between distance categories, as estimated by the model. The inverse logit function transforms the threshold (which represents a log-odds value) into a probability, ranging from 0 to 1. This transformation allowed for the interpretation of seal avoidance probabilities during signal emission. To determine whether adding a random intercept for individual seals improved the model fit, two models were compared using a likelihood ratio test (LRT): a more complex model incorporating both the fixed effect of ADS signal and a random intercept for the track ID, and a simpler model containing only the fixed effect of ADS signal without the random intercept.

## Results

**Fig. 4:**
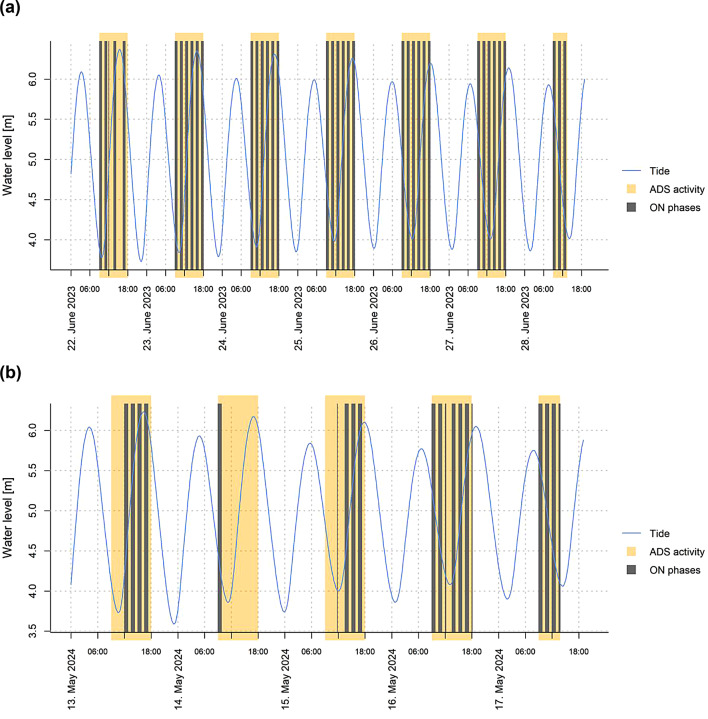
Water level (blue line) and ADS activity (orange highlighted) shown separately for the two trial periods: (**a**) trial period 1 and (**b**) trial period 2. The figure illustrates fluctuations in water levels alongside periods of ADS activity, with dark grey bars marking ADS signal ON phases.

During the first trial period, ON phases were generally evenly distributed (44% ON, 56% OFF) (Table 1; Fig. 4a). In contrast, the second trial period was misbalanced regarding ON and OFF phases (31% ON, 69% OFF) due to periods of battery recharging and the transducer unit not being fully submerged during extreme low-tide events. During these periods the ADS was turned off and did not emit any signal. In addition to the standard OFF phases without signal emission, such intervals were consequently analysed as OFF phases (Table 1; Fig. 4b).

**Table 1:** Total operational activity of the ADS during the two trial periods in hours and the corresponding percentual proportion in brackets of signal ON and OFF phases.

Trial period	Total operational activity	ON phases	OFF phases
1	58.50 h	25.90 h (44.27%)	32.60 h (55.73%)
2	40.83 h	12.61 h (30.88%)	28.22 h (69.12%)

In trial period 1, 18,150 seal positions were determined from 430 individual seal tracks. In the second trial period, 3,936 grey seal positions were detected from 79 seal tracks (Table 2).

**Table 2:** Summary of the still frame image dataset extracted from the timelapse video material recorded during the two trial periods. Further listing the number of images with at least one visible seal position as well as the number of recorded seal positions and reconstructed seal tracks.

Trial period	Extracted images	Images with seal positions	Recorded seal positions	Reconstructed seal tracks
1	105,300	9,961	18,150	430
2	73,500	3,276	3,936	79

**Fig. 5:**
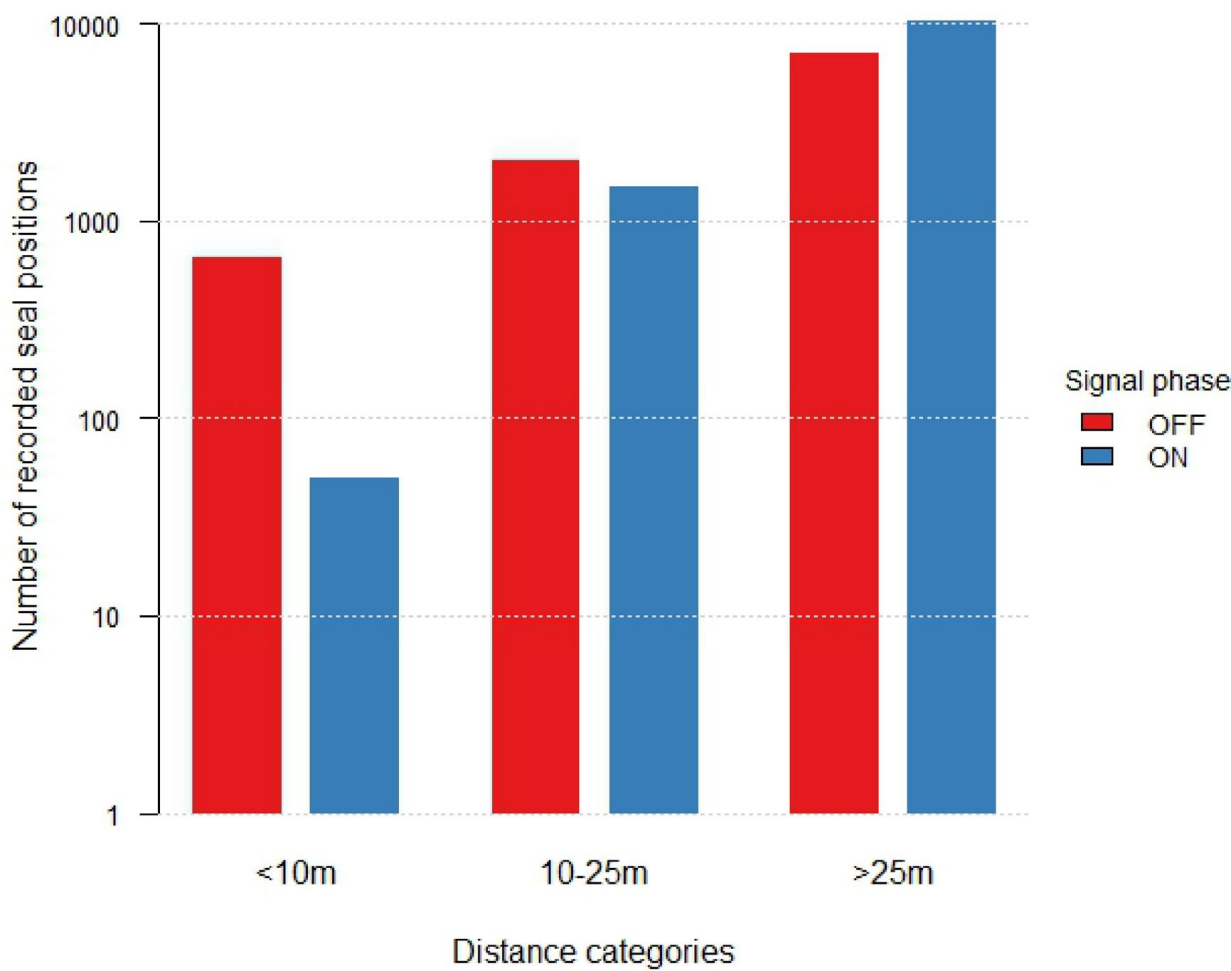
Distribution of the total number of recorded grey seal positions across three distance categories (< 10 m, 10–25 m, > 25 m) under different ADS signal conditions (OFF: red; ON: blue). The bar graph uses a logarithmic y-axis to enhance visualisation of data trends across ADS signal phases and distance categories.

Recorded grey seal positions were classified into three distance categories (< 10 m, 10–25 m, and > 25 m) and further categorised based on their occurrence during ADS signal ON or OFF phases. In the first trial period, during OFF phases, most seal positions were recorded beyond 25 m, with fewer in the 10–25 m zone and very few within the < 10 m zone. During ON phases, the proportion of positions beyond 25 m increased, while fewer positions were observed in the two closer zones (< 10 m and 10–25 m) (Table 3). A similar pattern was found in the second trial period. During OFF phases, most seal positions were recorded beyond 25 m, followed by the 10–25 m and < 10 m zones, respectively. Notably, during ON phases, no seal positions were observed within the < 10 m or 10–25 m zones, with all positions located beyond 25 m (Table 3). The combined findings from both trial periods show the same trend as observed in the individual trial periods. Seal counts within 10 m of the transducer unit are lower during ON phases compared to OFF phases. In the 10–25 m range, seal counts are more similar between both signal phases, but still a noticeable increase during OFF phases can be observed. The highest seal counts are observed for both signal phases at distances greater than 25 m, with a higher frequency when the signal is ON (Fig. 5). Track data provide an additional perspective on seal movement behaviour in relation to the ADS signal phase. The distribution of reconstructed tracks mirrors the spatial pattern observed in the positional data, with substantially fewer tracks recorded in close proximity to the transducer (< 10 m) during ON phases. Track frequency beyond 25 m remains high under both signal states and exhibits a slight increase during ON phases, supporting the interpretation of spatial avoidance behaviour during signal activity (Table 3).

**Table 3:** Distribution of reconstructed grey seal tracks and recorded positions across distance categories and by ADS signal phase during both trial periods. Tracks were counted for both trial periods based on the occurrence of seal positions within each distance category with tracks spanning multiple categories or signal phases counted in each relevant category. Position counts are shown separately for both trial periods and in total.

Distance category	ADS signal phase	Number of Tracks	Position count trial period 1	Position count trial period 2	Total position count
< 10 m	OFF	96	502	162	664
	ON	12	51	0	51
10–25 m	OFF	162	1,389	693	2,082
	ON	45	1,515	0	1,515
> 25 m	OFF	289	4,314	2,906	7,220
	ON	158	10,379	175	10,554
**Total**			**18**,**150**	**3**,**936**	**22**,**086**

The likelihood ratio test (LRT) showed that the inclusion of the random intercept for track ID significantly (p-value < 0.001) improved the model fit (AIC = 15,342; log-likelihood = −7,666.8) compared to a simpler model without the random intercept (AIC = 24,869; log-likelihood = −12,431.6), indicating that accounting for variability in seal behaviour during each track enhanced the model’s explanatory power. With the random intercept included, we found a significant effect of the ADS signal on spatio-temporal occurrence of grey seals. A comprehensive summary of the model parameters, including both log-odds estimates and exponentiated odds ratios with their 95% confidence intervals (CI) and p-values is presented in Table 4. The estimated coefficient for the ADS signal ON phase was 1.2735 (Std. Error = 0.208, z-value = 6.129, p-value < 0.001). This corresponds to an odds ratio of 3.57 (95% CI: 2.38–5.37), indicating that during ON phases, seals were over three and a half times more likely to be observed in a farther distance category (10–25 m and > 25 m) rather than in the reference category (< 10 m), at each transition between categories, compared to OFF phases. The threshold coefficients were estimated as −10.3059 (Std. Error = 0.6308, z-value = −16.34) for the boundary between < 10 m and 10–25 m, and − 7.0611 (Std. Error = 0.6237, z-value = −11.32) for the boundary between 10 and 25 m and > 25 m, representing the cut points between the distance categories on the log-odds scale. The negative thresholds imply that seals are more likely to be observed in farther categories during signal emission. When converted to cumulative probabilities, the first threshold coefficient corresponds to P(Y ≤ 10 m) ≈ 3.34e^−05^, indicating a negligible probability of seals being observed within 10 m. The second threshold for ≤ 10–25 m (i.e., < 10 m or 10–25 m) gives P(Y ≤ 10–25 m) ≈ 8.57e^−04^, still very low but higher than the ≤ 10 m probability. These probabilities align with the negative threshold coefficients, confirming seals avoid closer distances when the ADS signal is ON. The random intercept of track ID captured substantial variability in baseline behaviour among seal tracks (variance = 55.23, Std. Dev. = 7.432), reflecting the inherent differences in movement patterns. Notably, the random intercept for track ID explained 94% of the overall variance in seal distances (ICC = 0.94), underscoring the importance of accounting for individual events and animal differences when analysing factors influencing seal behaviour.

**Table 4:** Comprehensive summary of the results from the cumulative link mixed model (CLMM) for ADS signal effect (ON vs. OFF) on grey seal positional proximity. Presented is the fixed effect of the ADS signal status ON on grey seal positional proximity to the transducer across three ordered distance categories (< 10 m, 10–25 m, > 25 m). Results for the fixed effect include both the odds ratio with its 95% confidence interval (CI) and p-value, and the Raw estimate (log-odds) with its standard error. The category thresholds represent the cut points between the ordinal distance categories, corresponding essentially to the model intercepts, therefore presented only in the link function scale (log-odds). Track ID was included in the model as a random intercept to account for repeated measures within individual tracks. Statistically significant (*p* < 0.05) values are highlighted in italics.

Variable	Odds ratio	Lower 95% CI	Upper 95% CI	*p*-value	Estimate (log-odds) ± Std. Error
ADS signal ON	3.57	2.38	5.37	*< 0.001*	1.2735 ± 0.208
**Category thresholds**					
< 10 m|10–25 m	-	-	-	*< 0.001*	−10.3059 ± 0.6308
10–25 m|>25 m	-	-	-	*< 0.001*	−7.0611 ± 0.6237

## Discussion

This study evaluated the effect of a novel acoustic deterrent system (ADS) on grey seal positions in the Helgoland Dune swimming zone, a key tourist destination and vital grey seal habitat. Our results demonstrate that the ADS signal significantly influenced seal distance to the sound source, promoting separation between seals and humans in recreational areas. We found a significant relationship (p-value < 0.001) between ADS signal emission phases and an increased distance of recorded seal positions from the transducer. Seals were 3.57 times more likely to be observed in the two farther distance categories (10–25 m and > 25 m) during signal emission compared to silent control periods, indicating a strong local avoidance response. This deterrent effect was particularly pronounced in the closest distance zone (< 10 m), where the probability of observing seals during signal emission was negligible. Inter-event variability accounted for 94% of the variance in seal positions, indicating that most of the variation could be attributed to differences between events and possibly to individual seals, thereby highlighting the importance of including the random intercept in the model. This pronounced inter-individual variability could suggests that personality traits and individual tolerance thresholds may play a role as important as the acoustic signal itself in determining and shaping avoidance behaviour. However, as individual seals could not be reliably identified across different tracks and each track was treated as an independent observation belonging to a new individual seal, further research is needed to clarify how much individual differences influence the deterring power of the device. Consequently, repeated measurements of the same individuals may have biased the results if certain seals consistently exhibited stronger or weaker responses to the ADS signal. Beyond this variability, age-related factors could also play a significant role in determining responsiveness. Götz and Janik^29^ suggested that older seals may exhibit reduced sensitivity to acoustic deterrents due to hearing impairments, which increase startle response thresholds^42^. This raises the possibility that age might modulate the effectiveness of the ADS signal. Intriguingly, on the second day during our first trial period, the camera captured a group of three young grey seals displaying a distinct behavioural pattern: prolonged play and chasing within the study area (44 min), regardless of ADS signal emission. During this time, they primarily remained in the 10–25 m and > 25 m distance categories, with only one individual briefly entering the < 10 m zone twice before quickly retreating. This observation suggests that, in addition to possible hearing impairments, age-related behavioural differences could also influence the ability to respond to the ADS signal. Supporting this notion, Scheer^20^ found that subadult grey seals were more likely to approach swimmers in the Helgoland Dune’s south beach swimming zone, indicating a potential link between age and exploratory or playful behaviour. However, Scheer’s study acknowledged several limitations, such as the inability to reliably identify age class in more than 50% of the seals and the short study duration (10 days), which may have led to repeated approaches by the same individuals, potentially biasing age-related findings^20^. These observations highlight the importance of understanding individual behavioural responses in grey seals, particularly in high-interaction areas like the Helgoland Dune, where younger seals appear to approach swimmers more frequently. To address this complexities, long-term studies with robust individual identification methods are essential. Future research should employ longitudinal tracking to explore how individual and developmental factors, such as age-specific risk tolerance and exploratory behaviour affect responses to the ADS signal. A deeper understanding of behavioural variation, particularly in high-risk interaction zones, could ultimately lead to more effective management strategies.

An unintended randomisation of ON/OFF phases was applied in the second trial period due to technical (battery capacity) and environmental (tidal variations) constraints. While this resulted in an irregular deployment pattern, the statistically significant deterrent effect in the pooled dataset suggests the ADS remained effective despite these operational variations. These findings imply that the ADS may not require strict adherence to a predetermined ON/OFF pattern to maintain efficacy. This operational flexibility presents a particularly beneficial feature for marine deterrent systems as it suggests that effective performance can be maintained even when operations are interrupted. This could allow for target specific sound emissions only in cases of seal approaches to human swimmers. Furthermore, we believe that this unintended feature of our observation period might have a positive effect, in the sense of reducing the probability of habituation, a common concern when using deterrents for marine mammals^43^.

The distance categories in this study (< 10 m, 10–25 m, > 25 m) were selected based on sound propagation calculations conducted under shallow water conditions and the previously published startle threshold for grey seals of 159 dB re 1 µPa at 1 kHz^29^. This established 25 m as the maximum radius within which startle responses could be reliably expected in most individuals, while the 10 m boundary was chosen to represent an immediate zone of high sound intensity. The resulting three-tiered distance framework provided a robust, empirically grounded structure for evaluating seal responses. However, as Götz and Janik^30^ point out, the startle threshold may in fact be lower under certain conditions. The originally published threshold was established in controlled experiments using pure tone stimuli with captive grey seals, which may not fully reflect responses to more complex or broadband signals in natural settings. As such, the effective deterrent range in the field could potentially extend beyond 25 m. This suggests that the distance categories used here may represent a conservative estimate of the actual behavioural response range.

The deterrent effect observed in this study aligns with previous applications of the ADS in other marine environments, notably within fisheries and aquaculture, where it has proven effective in deterring phocid seals and reducing depredation^25,29–33^. While the former research primarily focused on safeguarding economic resources, our study represents a novel application by evaluating the ADS in a recreational setting with frequent close human-seal interactions. The motivation of grey seal presence on the Helgoland Dune differs from aquaculture sites, which can represent important foraging resources. We could show that the effectiveness is maintained in a different setting. Here, the primary objective shifts from resource protection to fostering human-wildlife coexistence. Consistent with prior findings, we observed that grey seals maintained significantly greater distances from the sound source during signal emission (p-value < 0.001), further substantiating the ADS’s deterrent efficacy on our targeted species. Furthermore, the effect was limited to distances of only 25 m, which still allows the animals to enter the bay and use their important haul-out sites which are located about 250–300 m from the transducer on both sides of the swimming area. This novel application broadens the ADS’s utility beyond mitigating economic conflicts to addressing direct conservation challenges in tourism hotspots, positioning it as a versatile tool for managing coastal areas with overlapping human and seal activity.

The findings of this study hold important ecological and conservation implications, particularly for promoting human-wildlife coexistence in shared habitats. By demonstrating the effectiveness of the ADS in deterring grey seals from approaching human-populated areas, this research presents a promising management tool that balances wildlife conservation with human safety. Unlike traditional non-lethal strategies, such as physical barriers or spatial zoning, which are difficult to enforce in dynamic coastal environments and pose entanglement risks^44,45^the ADS offers a flexible, non-invasive alternative that requires no physical habitat modification. Moreover, by selectively influencing seal behaviour within a defined deterrence radius while allowing them to remain present in surrounding areas, the ADS aligns with conservation principles that emphasise minimising human impact while preserving natural wildlife distributions and behaviours^46^. This targeted approach maintains ecological connectivity by ensuring seals can still access their habitat outside the immediate deterrent zone, supporting more natural movement patterns than complete exclusion methods would permit. In addition, the ADS is engineered to minimise the risk of auditory harm to both target and non-target species by precisely controlling the key acoustic parameters of its signal^25,30^. The frequency of the sound is chosen to specifically target the hearing range of grey seals while avoiding overlap with the more sensitive hearing ranges of species like harbour porpoises^27,30^. Furthermore, the amplitude, calibrated to trigger an avoidance response in seals through the startle reflex without causing physical harm or stress, and the short duration of each sound emission, are designed to minimise prolonged exposure and reduce the risk of both temporary and permanent hearing damage in both target and non-target speices^29,30,33^. This refined acoustic design results in a more sustainable and ethically responsible alternative to conventional deterrent devices^25,30,33^.

While our results confirm the ADS’s potential to promote human-wildlife coexistence in coastal areas, the limited experimental duration of the trial periods necessitates extended studies to evaluate the ADS’s reliability for sustained and permanent use in recreational settings. A critical consideration is thereby the potential for two opposing behavioural responses: habituation (a gradual decrease in response to repeated stimulus exposure) and sensitisation (an increased response with recurring exposure)^23^. For acoustic deterrents, habituation often poses a significant challenge, as animals may learn to reclassify the stimulus as non-threatening after repeated exposure without negative consequences^43^. In contrast, Götz and Janik^29^ demonstrated that eliciting the acoustic startle reflex in captive grey seals can induce sensitisation, amplifying flight and avoidance responses, even overriding food motivation. The ADS signal exploits this reflex through emission of band-limited noise pulses with rapid onset times^25,29^. While these findings are promising, the long-term behavioural impacts under natural conditions, particularly how human-seal interaction dynamics in shared coastal environments influence response persistence, remain unclear.

In this study, only a part of the swimming zone was exposed to sound levels exceeding the behavioural response threshold of 159 dB re 1 µPa^29^. While our findings demonstrate localised deterrence, the practicality of scaling this solution for effective, continuous coverage across the entire swimming zone is a key consideration. This would necessitate more extensive trials with multiple and coordinated transducers. These investigations should precisely determine the required number and optimal spatial layout of transducers to reliably achieve a dosed deterrence over larger areas. Such an assessment is essential to create sufficient coverage while carefully managing potential habituation and minimising disturbance. From a practical management perspective, the realistic deployment and long-term viability of the system depend on considerations such as robust power supply, secure anchoring solutions, ease of deployment and retrieval in dynamic conditions, and minimal interference with other human users (e.g., swimmers). Evaluating these operational aspects will be central to confirming the ADS’s overall feasibility and utility as a viable conservation and management tool in dynamic recreational settings.

As an initial field test of the ADS in a recreational context, this study was conducted early in the tourist season when swimmer presence was moderate compared to peak summer months. Furthermore, the sampling duration, as mentioned earlier, was limited, consequently affecting the number of distinct seal observation events. These temporal constraints may have influenced the strength and generality of the observed behavioural patterns. Furthermore, while tracking the total number of individual seals over time and accounting for haul-out size could have provided valuable context for interpreting deterrent effects, such measures were beyond the scope of this study. Accordingly, the results should be viewed as a proof of concept, demonstrating the feasibility and potential of targeted acoustic deterrence in a shared human-grey seal recreational area under real-world conditions. Future research should evaluate the system under conditions of higher human activity levels and over extended time periods to explore potential variation in seal responses, including whether the increased presence of swimmers during peak tourist seasons may add further motivation for grey seals to enter the area of sound exposure.

## Conclusion

The present study is the first to test the potential of a novel acoustic deterrent system (ADS) emitting startle-inducing sound signals as a deterrent for grey seals in shared coastal environments. Our results show that grey seal positions were significantly more often recorded in farther distance categories during signal emission compared to silent control periods. This effect remained consistent across varying environmental and operational conditions throughout the two trial periods, underscoring the system’s robustness for real-world use. Originally developed to protect fisheries and aquaculture from seal depredation, the ADS demonstrated to be a scalable, non-invasive solution to mitigate human-seal conflict in tourism hotspots, where balancing safety, animal welfare, and habitat preservation is essential. The findings of our study underscore its potential to foster safer human-wildlife coexistence in areas with frequent interactions. However, individual variation in behaviour and potential age-related differences emphasise the importance of continued research to ensure the ADS’s long-term effectiveness and robustness, especially in ecologically dynamic coastal settings.

## Supplementary Information

Below is the link to the electronic supplementary material.

Supplementary Material 1

## Data Availability

All data generated or analysed during this study are included in this published article and its supplementary information file.
